# GnRH analogs as a monotherapy in transgender and gender-diverse adolescents: clinical insights from a single-center study

**DOI:** 10.1530/EC-25-0292

**Published:** 2025-08-14

**Authors:** Fleur A H Lahaije, Petra A van Setten, Willemien Levels, Karlijn Becking-Malpasso, Hedi L Claahsen-van der Grinten

**Affiliations:** Department of Pediatrics, Amalia Children’s Hospital, Radboud University Medical Centre, Radboudumc Expert Center for Sex & Gender, Nijmegen, The Netherlands

**Keywords:** transgender, gender diverse, GnRH analogs, GnRHa, Pamorelin

## Abstract

**Background:**

Gonadotropin-releasing hormone agonists (GnRHas) are widely used in the treatment of transgender and gender-diverse adolescents to prevent the development of undesired physical changes. However, the safety of GnRHa use remains a subject of debate and objective literature on this topic is limited. In particular, there is a lack of studies comparing the effects of GnRHas at different Tanner stages, as the effectiveness of GnRHa treatment in adolescents who are close to completing puberty remains uncertain.

**Aim:**

The aim of this study was to evaluate the effects of GnRHa monotherapy in transgender adolescents with gender dysphoria (GD) at early versus late Tanner stages.

**Methods:**

This retrospective study analyzed the electronic medical records of adolescents with GD who were treated with GnRHa monotherapy at the Radboudumc Expert Center for Sex & Gender. Treatment duration ranged from 0.5 to 2 years, with follow-up every three months. The outcomes assessed included biometrics, biochemistry, and self-reported side effects.

**Results:**

The study included data from 67 assigned females at birth (AFAB) and 33 assigned males at birth (AMAB). A total of 51 adolescents were classified as Tanner stage 2 or 3, and 49 were classified as Tanner stage 4 or 5. 33% of the participants had psychiatric coexisting conditions, most commonly attention deficit hyperactivity disorder (19%) and ASD (18%). In addition, 36% of the adolescents were either overweight or obese. During follow-up, gonadotropin levels were not fully suppressed, particularly in the Tanner 4/5 group, while sex hormone levels were suppressed in nearly all adolescents. Side effects, especially hot flushes, abdominal discomfort, and emotional disturbances, were significantly more common in the Tanner 4/5 group, with 76% of this group reporting hot flushes. The impact of GnRHa treatment on pubertal development was minimal. Overweight and psychiatric comorbidities were prevalent among the adolescents.

**Conclusion:**

GnRHas effectively suppressed sex hormone levels in adolescents with GD, although gonadotropin suppression was not complete, particularly in the Tanner 4/5 group, where gonadotropin levels remained elevated. Side effects were frequently reported, particularly in the Tanner 4/5 group, while the impact on pubertal development was limited. Therefore, the benefits and drawbacks of GnRHa treatment should be carefully considered, particularly in adolescents at Tanner stages 4 and 5.

## Introduction

Gender incongruence (GI) is defined in the Diagnostic and Statistical Manual of Mental Disorders (DSM-5) as a condition in which an individual’s gender identity does not align with the gender assigned at birth. The World Health Organization defines ‘GI of Adolescence and Adulthood’ as ‘a marked and persistent incongruence between an individual’s experienced gender and the assigned sex, often leading to a desire to transition. This transition may involve hormonal treatment, surgery, or other healthcare services aimed at aligning the individual’s body, as much as desired and to the extent possible, with their experienced gender’ (ICD-11 Diagnostic code HA60). When GI is accompanied by psychological symptoms, it is referred to as gender dysphoria (GD). The reported prevalence of GD in childhood and adolescence ranges from 0.6 to 1.7% (1). However, the number of transgender and gender-diverse adolescents (TGDAs) seeking hormonal treatment continues to rise in many countries, particularly among individuals assigned female at birth (AFAB) (2, 3, 4). The reasons for this significant increase in prevalence and the skewed sex distribution remain unclear. It is generally suggested that heightened societal awareness and acceptance play key roles, making TGDAs more visible and motivating them to seek assistance (2, 5).

The World Professional Association for Transgender Health (WPATH) and the Endocrine Society have established specific guidelines for the diagnosis and treatment of TGDAs (6). In addition, many European countries have developed quality standards that outline care for TGDAs within their specific contexts. In summary, all adolescents must undergo a thorough assessment by a specialized team, which should include a qualified mental health provider, a pediatric endocrinologist, and, preferably, a gynecologist who can address the effects of hormonal treatment on reproduction. Furthermore, existing guidelines stipulate that any coexisting psychiatric conditions should be addressed. The decision to initiate hormonal treatment is always made by a multidisciplinary team.

For TGDAs under the age of 16 who have already entered puberty, gonadotropin-releasing hormone analogs (GnRHas) may be offered to prevent further unwanted pubertal development. Suppression of secondary sex characteristics can alleviate dysphoric symptoms and related psychological distress (7). GnRHas, administered intramuscularly, suppress the production of gonadotropins, including luteinizing hormone (LH) and follicle-stimulating hormone (FSH), thereby reducing endogenous sex steroid hormone production (8). This treatment, known as the ‘Dutch Protocol,’ was introduced nearly 30 years ago (9). It is considered fully reversible, as the production of sex hormones resumes after discontinuation of the treatment. Several studies have shown improvements in GD and reductions in depression and improved psychological functioning in TGDAs (10, 11, 12). Research has demonstrated that TGDAs treated with GnRHas are unlikely to discontinue treatment during monotherapy, with most continuing with gender-affirming hormonal therapy by the age of 16 (13, 14).

While the primary goal during early puberty is to prevent the development of undesired secondary sex characteristics and provide time for further evaluation, GnRHa use in late puberty may serve as a temporary intervention to ‘buy time,’ particularly when gender-affirming hormones cannot yet be initiated due to age or psychosocial factors. However, in late-pubertal TGDAs, the benefits of GnRHas should be carefully weighed against potential side effects, especially since the desired outcomes – particularly regarding physical changes – may be more limited. Therefore, comprehensive and individualized counseling is crucial when considering GnRHa treatment for this group.

There is a lack of studies addressing the short- and long-term complications associated with the initiation of GnRHa treatment during early puberty, particularly with prolonged suppression of sex hormones during this critical period of growth and psychosexual development. Furthermore, ongoing political and scientific debates about the efficacy of this medication persist, especially regarding children with late-onset GD and psychiatric comorbidities (15), with some countries opting to restrict pediatric transgender care. Criticisms include the reliance on historical cohorts in many studies, the absence of long-term follow-up, the lack of control groups, and poor methodology in some research (15, 16). In contrast to childhood-onset GD, which typically manifests during early childhood, late-onset GD emerges in adolescence or adulthood, often following the development of secondary sexual characteristics or after significant life events. The National Institute for Health and Care Excellence (NICE) has recently rated the quality of the evidence as very low (17). A recent review summarized concerns regarding GnRHa treatment (18). However, other papers criticize the use of existing data and the findings of the Cass report on GnRHa use (19, 20).

In addition, current guidelines recommend starting GnRHa treatment only after the onset of endogenous puberty (Tanner stage 2/3). There is limited evidence on the positive effects of GnRHas in adolescents who have reached advanced puberty (Tanner stage 4/5). In this group, only minimal positive effects are expected, particularly concerning physical changes such as voice deepening or penis size in individuals assigned male at birth (AMAB) or breast development in those assigned female at birth (AFAB).

The aim of this study is to investigate the short-term effects of GnRHa monotherapy in TGDAs within a single-center setting. We evaluated the impact of GnRHa treatment on three domains: biometrics, biochemistry, and adverse effects. We compared these parameters in TGDAs starting treatment at Tanner stage 2/3 with those starting treatment at Tanner stage 4/5.

## Patients and methods

We performed a retrospective, descriptive study using the electronic records of TGDAs treated at the Radboudumc Expert Center for Sex & Gender (REGG), starting from March 2020. Data were collected from the electronic files and were analyzed anonymously. Due to the retrospective design of the study, no ethical approval or written informed consent was necessary. General data were collected at baseline. Data on different biometric and biochemical parameters were collected before the start of GnRHa treatment and every three months following the start (T0 = baseline, T1 = 3 months, T2 = 6 months, etc.).

### Patients

**Figure 1:**
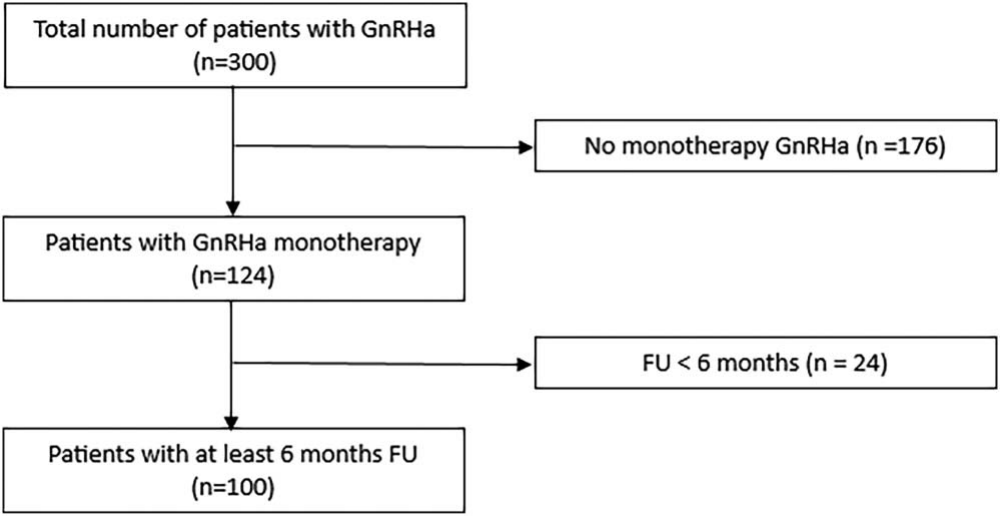
Flow chart of inclusion.

We included adolescents below the age of 18 years treated with GnRHa monotherapy after they were diagnosed with GD by a qualified and experienced team of psychologists. The follow-up period was at least 6 months after the start of the treatment. Adolescents with insufficient follow-up data or adolescents who were also treated with gender-affirming hormonal treatment were excluded (Fig. 1). The included TGDAs were divided into groups: assigned female at birth (AFAB) and assigned male at birth (AMAB). Furthermore, the adolescents were divided based on the Tanner stage at the start of the treatment: Tanner stage 2/3 and Tanner stage 4/5.

All adolescents were treated following a local protocol based on national and international guidelines (21): criteria for the start of GnRHa treatment in the REGG include at least Tanner 2, no medical or psychological contraindications, confirmed diagnosis of GD after careful assessment by the psychological team, written informed consent from the adolescent and parents, and fertility counseling by an experienced fertility doctor. All adolescents are discussed in a multidisciplinary (MD) meeting, and decisions about treatment are made within the MD team. The GnRHa given was 11.25 mg Pamorelin®, intramuscularly injected every 10–12 weeks. Clinical follow-up was performed every three months, including psychological follow-up.

## Outcome measures

### Physical examination

Biometric variables, including weight, height, and blood pressure, were collected at baseline. Weight was measured using an electronic floor scale (Seca 704, Seca Benelux, Belgium) in kg accurate to 0.1 kg. Height was measured using an electronic stadiometer (Seca 222, Seca Benelux). Body composition was divided based on body mass index (BMI) into underweight (weight <−1SD), normal (weight: −1SD to 1SD), overweight (weight >1SD), and obesity (weight >2SD). These definitions are in line with the current Dutch guidelines (22).

Blood pressure (systolic blood pressure (SBP) and diastolic blood pressure (DBP)) was measured at rest (three times) using an automated blood pressure monitor (Dinamap Carescape V100, GE Healthcare, Netherlands). Mean SBP and DBP were calculated from three repeated measurements. The upper value for normal blood pressure was defined as systolic and diastolic pressures below the age-based 90th percentile.

Tanner stages for breast, external genitalia, and pubic hair were determined at T0 by experienced examiners and were categorized from Tanner stages 1 (no pubertal development) to 5 (fully developed). Axillary hair was also categorized from Tanner stages 1 to 5, and a mean level of Tanner stage was reported. Testis volume was determined using a Prader orchidometer. Information about Tanner stages at later time points was self-reported. Self-reported Tanner staging is based on two methods: i) pictures of the genitals with different Tanner stages and ii) using the orchidometer by the individuals themselves after instruction by the health professional.

### Laboratory tests

All blood samples were drawn before the start of the treatment (T0), after 6 months (T2), and thereafter annually at different time points of the day. Blood samples were matched to the nearest time point. The following variables were measured:

- Hormone levels: testosterone was measured by liquid-chromatography tandem mass spectrometry (LC-MS/MS). Estradiol was measured in serum/plasma using a c8000 random access analyzer (Roche, Germany/Switzerland). LH and FSH were measured by serum using immunoassay using an E801 random access analyzer (Roche).
- Renal function: estimated glomerular filtration rate (eGFR).
- Liver function: alanine transaminase (ALAT), aspartate aminotransferase (ASAT), lactate dehydrogenase (LDH), and gamma-glutamyl transferase (gamma-GT).
- Blood count: hemoglobin, hematocrit, leukocytes, and thrombocytes.
- Bone health: vitamin D.

### Self-reported side effects

During every visit, the adolescents were asked about side effects following a standardized list of symptoms. Variables for self-reported side effects were documented every three months and extracted from electronic files. The adolescents were asked open-ended questions if they experienced side effects. Side effects were categorized into three categories by the leading PI based on the descriptive data: no side effect, mild (no burden on daily life), and severe (burden on daily life). Spotting, defined as a single episode of bleeding after the initiation of GnRHa treatment, is a well-recognized and expected phenomenon and is therefore not considered a side effect in this context.

### Statistics

All data were analyzed using IBM SPSS statistics 27. Descriptive statistics were used to calculate frequencies and means of subgroups for the description of the population. Continuous variables were reported as mean and range (differences between minimum and maximum values). Categorical or dichotomous variables were reported as number and percentage. Changes in biochemistry between the Tanner 2/3 and Tanner 4/5 subgroups were analyzed using one-way ANOVA. Side effects across different time points were compared between Tanner 2/3 and Tanner 4/5 using the chi-square test.

## Results

### Baseline characteristics and biometrics

In total, 67 AFAB and 33 AMAB were included in this study and were evaluated for at least six months from the start of the treatment with the GnRHa. Table 1 shows the baseline characteristics. The mean age at the start of the treatment was 12.7 years in AFAB and 13 years in AMAB. Besides height, which was significantly higher in AFAB, there were no significant differences in biometrics between both groups. 35.8% of AFAB and 36.4% of AMAB were, respectively, classified as overweight and obese at the start of the treatment. In the AFAB group, 30 (44.8%) were classified as Tanner stage 2/3 and 37 (55.2%) were Tanner stage 4/5 at the start of the treatment. In the AMAB group, 21 (63.6%) were classified as Tanner stage 2/3 and 12 (36.4%) were Tanner stage 4/5 at the start of the treatment.

**Table 1:** Baseline descriptive variables of 100 TGDAs at the start of GnRHa treatment.

	AFAB	AMAB	*P*
	(*n* = 67)	(*n* = 33)	
Age (years), mean (range)	12.7 (10–16)	13.0 (11–18)	0.39
Biometrics			
Height (m), mean (range)	1.61 (1.47–1.85)	1.67 (1.48–1.86)	**0.005**
Weight (kg), mean (range)	53.7 (32.8–85.2)	55.7 (36.1–111.2)	0.53
BMI SDS, mean (range)	0.66 (−2.38–3.06)	0.52 (−2.20–3.97)	0.62
Overweight/obesity, *n* (%)	24 (35.8)	12 (36.4)	0.96
Systolic blood pressure (mmHg), mean (range)	117.3 (97–142)	118.4 (90–157)	0.67
Diastolic blood pressure (mmHg), mean (range)	65.0 (43–78)	63.7 (50–88)	0.41
Pubertal development			
Menarche (%)	35 (52.2)	-	-
Testicular volume left (mL), median (range)	-	10 (3–20)	-
Testicular volume right (mL), median (range)	-	10 (3–20)	-
Tanner stage			0.08
Tanner 2/3, *n* (%)	30 (44.8)	21 (63.6)	
Tanner 4/5, *n* (%)	37 (55.2)	12 (36.4)	
Comorbidities			
Psychiatric comorbidity overall, *n* (%)	20 (29.9)	13 (39.4)	0.34
ADHD/ADD, *n* (%)	12 (17.9)	7 (21.0)	0.69
ASD, *n* (%)	11 (16.4)	7 (21.0)	0.56
Depression, *n* (%)	2 (3.0)	0 (0.0)	0.32
Anxiety, *n* (%)	2 (3.0)	1 (3.0)	0.98
Psychiatric medication use, *n* (%)	9 (13.4)	9 (27.3)	0.09
Lifestyle factors			
Smoking, *n* (%)	1 (1.4)	1 (3.0)	0.28
Alcohol, *n* (%)	2 (3.0)	1 (3.0)	0.97
Drugs, *n* (%)	1 (1.4)	0 (0.0)	0.49
Dairy intake, mean (range)	2.17 (0–3)	2.20 (0–4)	0.92
Physical activity (*n* = 77)			0.29
Limited, *n* (%)	7 (13.2)	5 (20.8)	
Moderate, *n* (%)	23 (43.4)	13 (54.2)	
Intense, *n* (%)	23 (43.4)	6 (25.0)	

Bold indicates statistical significance. Abbreviations: TGDAs, transgender and gender-diverse adolescents; GnRHa, gonadotropin-releasing hormone agonist; AFAB, assigned female at birth; AMAB, assigned male at birth; kg, kilogram; BMI, body mass index; mmHG, millimeter of mercury; ADHD/ADD, attention deficit hyperactivity disorder/attention deficit disorder; and ASD, autism spectrum disorder.

29.9% of the AFAB and 39.4% of the AMAB reported any psychiatric comorbidity; attention deficit hyperactivity disorder (ADHD)/ADD and autism spectrum disorder (ASD) were most frequently seen in both groups. 13% of AFAB and 27% of AMAB used psychiatric medication. Smoking, alcohol use, and drug use were low in both groups (3% or less), which might be explained by the younger age groups. 13% of AFAB and 21% of AMAB reported limited physical activity.

### Pubertal development

In AFAB, 30 adolescents started GnRHa treatment at Tanner 2/3 and 37 at Tanner 4/5 (Table 1). As anticipated in the Tanner 2/3 group, in none of these subjects, menarche occurred before the start of the treatment. Three AFAB reported spotting within the first 3 months after the start of GnRHa treatment. In the Tanner 4/5 group, 35 of 37 (94.6%) AFAB with Tanner 4/5 had reached menarche prior to GnRHa treatment, of which 18 (49%) used progestogens to suppress their menstrual cycle before the start of the treatment. Three months after the start of the treatment (T1), 5 persons (14%) still had menstruation and 4 persons reported spotting. At T2 and T4, no menstruation was reported, and at T3, one person reported spotting. Pubertal development was effectively suppressed in both Tanner 2/3 and Tanner 4/5 groups. However, these adolescents self-reported no significant reduction in pubertal development during treatment.

In AMAB, 21 adolescents started GnRHa treatment at Tanner 2/3 and 12 at Tanner 4/5 (Table 1). Pubertal development did not significantly reduce during treatment in Tanner groups 2/3 and 4/5, nor was there any progression in pubertal development by self-reporting.

### Gonadotropin levels

**Figure 2:**
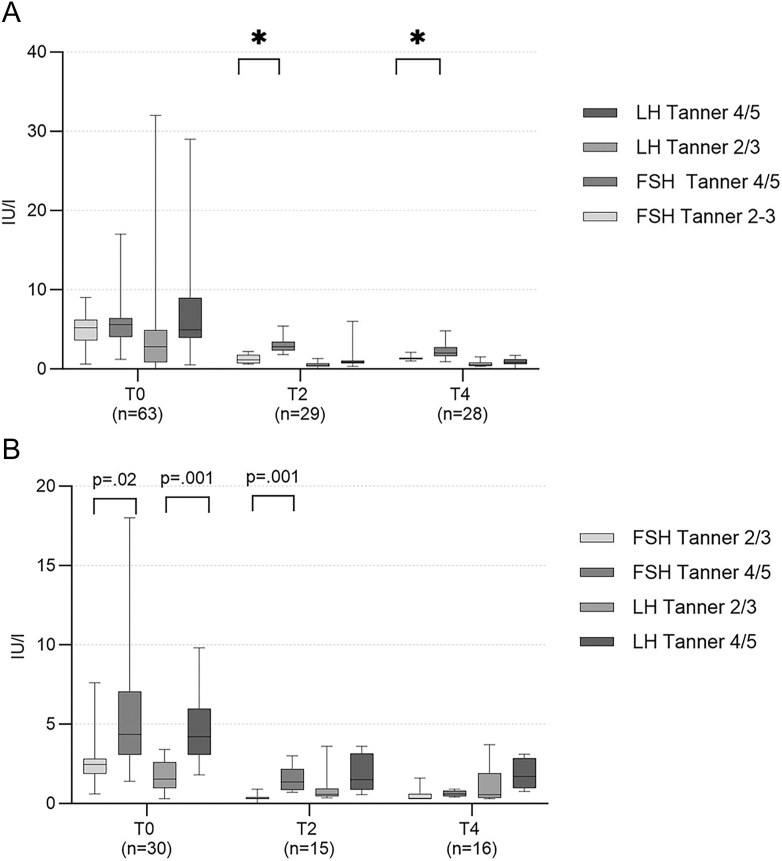
Gonadotropins over time in (A) AFAB and (B) AMAB.

Figure 2A and B shows the gonadotropin levels over time in AFAB and AMAB after a follow-up of 1 year.

In AFAB, the FSH levels showed significant differences between Tanner 2/3 and Tanner 4/5 groups at time points T0, T2, and T4. The Tanner 4/5 group had significantly (*P* < 0.001) higher levels of FSH (mean: 2.92 IU/L) compared with Tanner 2/3 (mean: 1.23 IU/L) at T2 and T4 (mean: 2.25 versus 1.37 IU/L, *P* = 0.008), respectively. The LH levels of the Tanner 4/5 group were also higher at different time points, compared with Tanner 2/3, although the differences were not significant (*P* = 0.20, *P* = 0.12, *P* = 0.12, at T0, T2, and T4).

In AMAB, FSH levels were significantly higher at T0 (*P* = 0.02) and T2 (*P* = 0.001) in the Tanner 4/5 group (5.98 and 1.53 IU/L, respectively), compared with Tanner 2/3 (2.91 and 0.35 IU/L, respectively). LH levels were also higher in Tanner 4/5 compared with Tanner 2/3 across all time points; however, only at T0, a significant difference was found (in Tanner 4/5 4.9 IU/L versus 1.78 IU/L in Tanner 2/3, *P* < 0.001).

### Sex steroid levels

**Figure 3:**
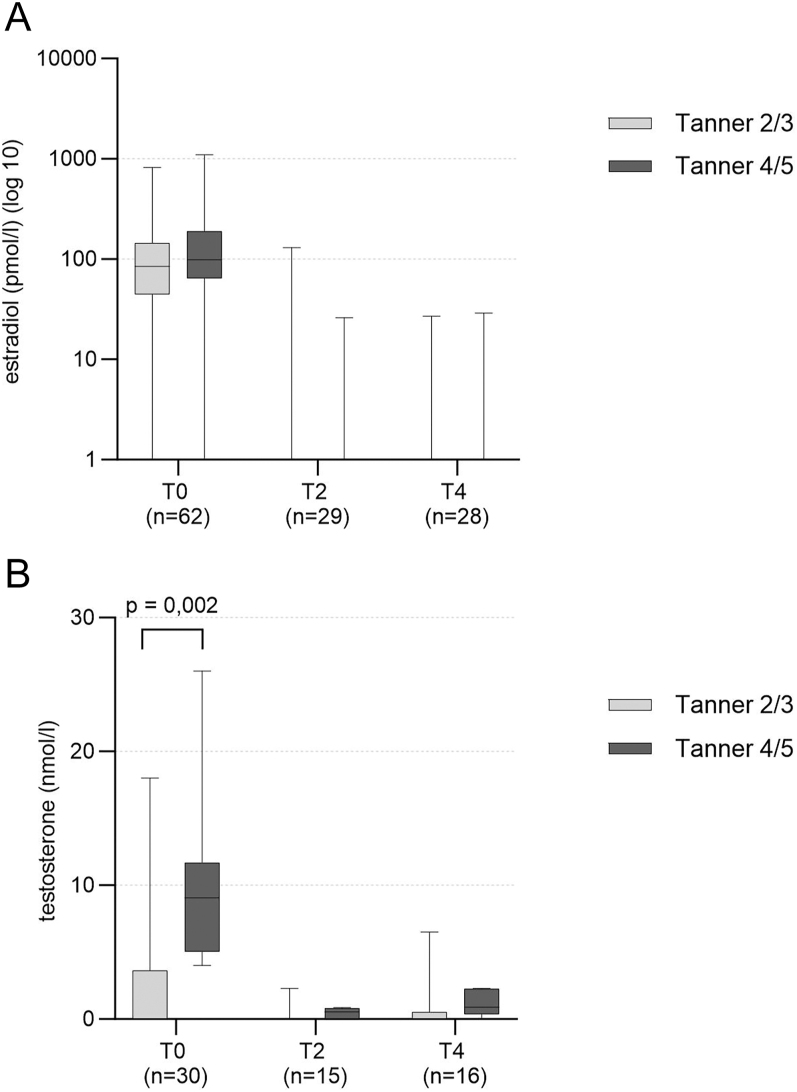
(A) Estradiol over time in AFAB. (B) Testosterone over time in AMAB.

Figure 3A and B shows the estradiol levels over time in AFAB and testosterone levels over time in AMAB.

In AFAB, estradiol levels significantly decreased below the reference levels for prepubertal individuals of the biological sex within the first 6 months of treatment. No significant differences between estradiol levels were found across any of the time points between Tanner 2/3 and Tanner 4/5 groups.

In AMAB, testosterone levels significantly decreased below prepubertal reference levels within the first 6 months of treatment. We found significantly higher (*P* = 0.002) testosterone levels in the Tanner 4/5 group (mean: 9.72 nmol/L) versus Tanner 2/3 (mean: 2.96 nmol/L) only at time point T0.

### Other laboratory tests

No abnormalities were found at baseline and after the start of the treatment in renal function, liver function, blood count, and vitamin D levels although some mild changes were detected between the Tanner 2/3 and Tanner 4/5 groups, which were clinically not relevant (Supplemental Tables (see section on Supplementary materials given at the end of the article)).

In AFAB, the Tanner 4/5 group had statistically significantly higher hemoglobin levels, hematocrit levels, lower urea levels and higher creatinine levels, lower AST, lower LDH, higher GGT, and lower vitamin D levels compared with Tanner 2/3.

In AMAB, the Tanner 4/5 group had significantly higher hemoglobin levels, hematocrit levels, creatinine levels, lower LDH, and higher GGT levels compared with Tanner 2/3.

### Side effects

Both, AFAB and AMAB experienced side effects after the start of the treatment. These side effects were experienced in both groups. Frequently reported side effects include flushing, fatigue, local side effects, abdominal complaints such as nausea and stomach aches, and emotional effects such as sadness and mood swings. Rare side effects (experienced once or twice) include concentration difficulties, floaters, hunger, hyperventilation, vertigo, dry or porous skin, pruritus, stamina decrease, tantrums, sleeping difficulties, epistaxis, palpitations, fainting, and pain in joints, scrotum, chest, and inguinal area.

**Figure 4:**
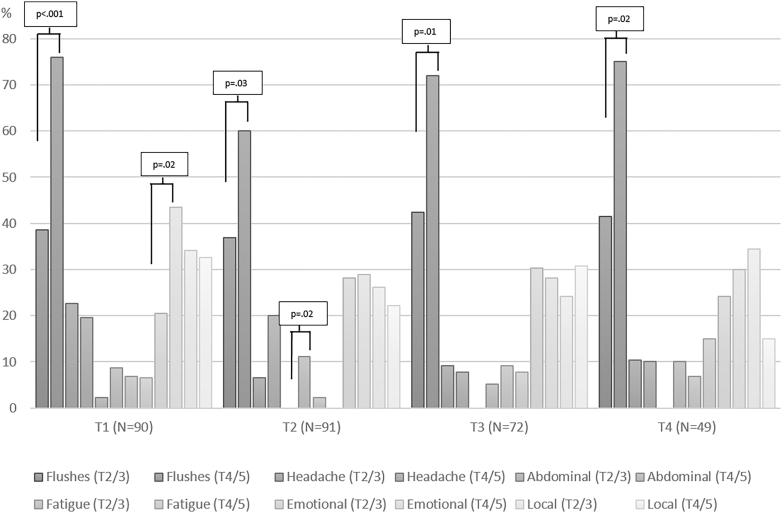
Side effects across time for Tanner stage 2/3 compared with Tanner stage 4/5.

Figure 4 shows the reported side effects in the Tanner 2/3 and Tanner 4/5 groups. Across all time points, a significantly higher percentage of adolescents in the Tanner 4/5 group reported flushes when compared to the Tanner group 2/3. At T1, 76% of Tanner 4/5 reported flushes, compared with 38.6% of Tanner 2/3. At T2, 60% of Tanner 4/5 versus 36.9% of Tanner 2/3 and, at T3, 72% of Tanner 4/5 compared with 42.4% of Tanner 2/3 in the Tanner 4/5 group experienced emotional problems (sadness, mood swings, and/or suicidality), compared with 20.5% in the Tanner 2/3 group (*P* = 0.02). At T2, 11.1% of the Tanner 4/5 group reported abdominal problems (nausea and/or stomach ache), compared with 0% in the Tanner 2/3 group (*P* = 0.02).

## Discussion

To the best of our knowledge, this is the first study to examine the short-term biochemical effects of GnRHa monotherapy in transgender adolescents within a large, recently treated cohort in a single-center setting. Our cohort of 100 individuals is relatively substantial, particularly for a single-center study conducted over a 3-year inclusion period. Several previous studies in this field have reported on smaller samples or used larger numbers over much longer periods (11, 14). We were able to study a significant number of transgender adolescents who received treatment according to both national and international guidelines in an academic setting, following a similar protocol. All adolescents in our cohort are treated under the same protocol, and both clinical and biochemical parameters were available for assessment. Our findings are relevant to the broader transgender population, as we included all adolescents who started GnRHa treatment, and we provide new insights into the effectiveness of GnRHas in more advanced Tanner stages.

The Dutch Protocol is often regarded as the gold standard for the treatment of TGDAs. In this protocol, the first step in hormonal treatment involves administering the GnRHa to suppress endogenous sex hormone production in adolescents younger than 16, regardless of Tanner stage. Adolescents aged 16 and older are eligible to begin treatment with gender-affirming hormones once a diagnosis of GD has been confirmed. According to the current Dutch quality standards, a minimum of 6 months of treatment with the GnRHa is required before starting gender-affirming hormonal treatment (21). However, the Dutch Protocol, particularly the use of GnRHas during adolescence, is not without controversy due to the non-negligible long-term risks associated with GnRHas, especially concerning bone health (23, 24).

### The clinical effects of GnRHas in late puberty on unwanted physical changes in Tanner stage 4/5 are limited

In our study, the mean age of AFAB was 12.7 years and the mean age of AMAB was 13.0 years. The majority of participants had already reached Tanner stage 4/5 at the start of GnRHa treatment. One previous study on puberty suppression reports an even older mean age of 14.75 years for all participants (11). No significant changes in Tanner stages were observed in adolescents treated with GnRHas, in both the Tanner 2/3 and Tanner 4/5 groups for both AFAB and AMAB individuals. In our AFAB group, no regression of breast tissue development is noted, although regression has been previously described in earlier Tanner stages (8). Another study indicates that the earlier the onset of suppression, the greater the effects on preventing breast development, finding only limited effects when GnRHas were started in Tanner 4/5. This study also shows little difference in the number of mastectomy surgeries in this group compared with controls (25).

In AMAB, individuals with Tanner stage 4/5, GnRHa treatment may result in less noticeable body hair growth, which could be considered a benefit of GnRHa treatment. However, the effect of GnRHas on body hair growth is difficult to quantify and could likely be achieved through less systemically impactful methods, such as electrolysis. That said, it is important to note that alternative options for suppressing testosterone are limited. Anti-androgens may also be used for this purpose but are associated with risks, such as hepatotoxicity (26), hyperprolactinemia, and meningiomas (27), while GnRHas are generally considered safer and more effective for suppressing testosterone during feminizing treatment (28).

Although we did not assess psychosocial outcomes in this study, the existing literature suggests that the psychosocial benefits of GnRHa treatment are less pronounced in adolescents who have already developed secondary sexual characteristics compared with those who are in earlier stages of puberty (29). Therefore, GnRHa treatment in Tanner stage 4/5 offers fewer benefits, but the negative effects on bone mineral density and other side effects still occur. Bone density typically decreases during GnRHa treatment; however, the literature suggests that at least partial recovery of bone density occurs after treatment is stopped (30).

Consequently, we recommend that especially AFAB adolescents in Tanner stage 4/5 be counseled on the limited effectiveness of GnRHa treatment in these stages, as well as the prominence of side effects. AFAB individuals experiencing dysphoria due to menstruation should be informed of alternative pharmacological options for suppressing their menstrual cycle, such as oral contraceptives, hormonal intrauterine devices (IUDs), and progestogens. These options are generally well tolerated, less invasive, and carry fewer negative effects on bone mineral density when used for limited periods. However, progestogens and oral contraceptives should only be prescribed after careful counseling about potential risk factors. Estrogen-containing contraceptives are associated with an increased risk of venous thromboembolism and arterial events (31, 32), while progestogen-only regimens may carry metabolic and mood-related side effects (33).

Our findings demonstrate that GnRHa treatment effectively suppresses sex hormone levels, but it does not completely suppress gonadotropins, particularly in adolescents at Tanner stage 4/5. This contrasts with a previous study that found higher estradiol levels in AFAB adolescents at Tanner 4/5, with gonadotropin suppression, although treatment protocols differed slightly, such as the use of short-acting Decapeptyl compared with the long-acting GnRHa used in our protocol (administered every 3 months). Therefore, LH and FSH are not reliable parameters for assessing the effectiveness of GnRHa treatment.

### GnRHa treatment should be prescribed with careful consideration in adolescents at late stages of puberty

Our study reveals that starting GnRHa treatment during late puberty (Tanner stage 4/5) leads to a significantly higher prevalence of side effects compared with adolescents starting treatment at earlier Tanner stages. This may be due to a sudden drop in sex hormone levels, leading to symptoms associated with a hypogonadal state. Reported side effects are comparable to what other studies found (34, 35). While an earlier study suggests that hot flashes only occur early in treatment (36), we did not observe a significant decrease in the frequency of reported side effects, including hot flashes, over time in either AFAB or AMAB adolescents. In contrast, starting treatment during early puberty (Tanner stage 2/3) prevents unwanted pubertal development but typically requires a longer treatment duration before initiating gender-affirming treatment. This extended period may lead to more long-term complications, particularly related to hypogonadotropic hypogonadism during the natural puberty period, such as a decrease in bone density, as noted in several previous studies (23, 37).

### Biological puberty facilitates fertility preservation

Due to long waiting lists, many adolescents in our study had already reached Tanner stage 4/5 by the time they started GnRHa treatment. A notable proportion of AFAB adolescents had already experienced menarche (49%). In AMAB adolescents, the mean testicular volume at the start of GnRHa treatment is >9 mL. One advantage of starting treatment at a more advanced Tanner stage, particularly in AMAB adolescents, is that it facilitates fertility preservation. A certain progression of puberty is required for semen preservation, as spermarche must have occurred. Spermarche typically takes place at Tanner stage 3, with a mean testicular volume of 11.5 mL (38). In addition, testicular volume correlates with sperm concentration and the percentage of motile sperm (39). Later stages of puberty facilitate semen preservation, but this progression carries the risk of further virilization, which may not be acceptable for the adolescent.

### Lifestyle factors and psychiatric comorbidity contribute to long-term outcomes

Our study cohort exhibits a high prevalence of comorbid conditions. A significant proportion of adolescents in our study are overweight, often coupled with insufficient physical exercise. These findings corroborate previous studies, indicating a high prevalence of overweight and limited physical exercise among TGDAs (40). Many TGDAs feel uncomfortable or self-conscious about their bodies, which can make physical exercise challenging. For example, the use of binders in AFAB adolescents can obstruct breathing, thus impeding optimal participation in physical activities. Other factors, such as changing in public locker rooms or being unable to participate in teams of the preferred gender, may also play a role. Moreover, TGDAs are at increased risk of disordered eating, often as a coping mechanism for GD (41). Therefore, emphasizing a healthy lifestyle, including adequate calcium intake, vitamin D, and physical exercise, remains important in the standard care and follow-up of these adolescents, especially considering the known impact of GnRHas on bone mineral density.

In our cohort, we observe a high percentage of psychiatric comorbidities, particularly neurodivergent conditions such as autism spectrum disorder (ASD) and ADHD. These findings align with previous studies (42, 43, 44). Psychiatric medications were frequently used in our population, especially among AMAB adolescents (27.3%), but also among AFAB adolescents (13.4%), compared to approximately 4% in the general adolescent population in the Netherlands (45, 46). Although the literature suggests that TGDAs are at increased risk of substance use disorders (47), we did not observe a high percentage of substance use, possibly due to the younger age of our participants. Given the common presence of psychiatric comorbidity, which may mask other conditions mimicking GD, a thorough diagnostic process remains essential. Adolescents should be supported by appropriate psychiatric care during treatment, with mental health professionals involved in follow-up care. In addition, standard care should be adapted to meet the specific needs of TGDAs with psychiatric comorbidities or neurodivergent conditions.

### A skewed sex ratio

Our study population included significantly more AFAB adolescents than AMAB adolescents (67 versus 33%). This ratio is consistent with the findings in earlier studies both in the Netherlands and abroad (3, 48, 49). This skewed sex ratio appears to be particularly pronounced in adolescents who present to gender clinics at older ages (50). Proposed explanations for this imbalance include the possibility that AFAB individuals are more likely to ‘catch up’ in terms of their gender identity expression, as well as increased visibility of transgender individuals in the media and shifting sociocultural factors influencing referral patterns (51). Another hypothesis is that it is more socially accepted for AFAB adolescents to express their gender identity compared with AMAB adolescents (52). However, a definitive explanation for this phenomenon remains unclear.

### Limitations

Our study has several limitations. Physical changes and Tanner stages were largely self-reported by the adolescents to minimize patient burden, as many find repeated physical examinations distressing. Side effects were also self-reported, and although subjective experiences are important, recall bias may have influenced these reports. While we included self-reported measures of pubic hair development, we did not follow up on overall body hair growth, which could impact AMAB patients’ satisfaction with GnRHa treatment. In addition, we experienced a reduction in the number of participants at later time points due to the initiation of cross-hormone treatment, leading to smaller groups of adolescents with advanced Tanner stages and reducing statistical power. Psychological side effects could be influenced by coexisting conditions, but as physiological side effects of GnRHas were our primary focus, we did not analyze mood and behavioral problems in relation to psychological comorbidities. More research into the subject is needed.

### Conclusion

In conclusion, our study shows that GnRHa treatment effectively suppresses sex hormone levels in both AFAB and AMAB adolescents. However, it is associated with a high prevalence of side effects that do not improve over time, particularly in adolescents with advanced puberty. Furthermore, it has limited effects on physical changes in late puberty. As a result, GnRHa treatment in late puberty should not be offered as standard care and should only be administered after thorough counseling about its potential advantages and disadvantages. Gonadotropins are not reliable markers for assessing GnRHa treatment effectiveness. More research on this topic is needed. Moreover, due to the high prevalence of somatic and psychiatric comorbidities, specialized care is essential for the follow-up of these adolescents. Therefore, we recommend that GnRHa monotherapy be administered only in specialized centers that implement a multidisciplinary approach with regular follow-ups.

## Supplementary materials



## Declaration of interest

The authors declare that there is no conflict of interest that could be perceived as prejudicing the impartiality of the work reported.

## Funding

This work was conducted without any financial support from external funding bodies, public or private.

## References

[1] Claahsen-van der Grinten H, Verhaak C, Steensma T, et al. Gender incongruence and gender dysphoria in childhood and adolescence-current insights in diagnostics, management, and follow-up. Eur J Pediatr 2021 180 1349–1357. (10.1007/s00431-020-03906-y) 33337526 PMC8032627

[2] Das E, Wasserbauer M, Loopuijt C, et al. Mijn gender, wiens zorg? Onderzoek naar de toename in en veranderingen van de vraag naar transgenderzorg [My gender, whose care? Research on the increase and changes in demand for transgender care]. Radboud University and Radboudumc research report. The Hague, Netherlands: ZonMw, 2023. (https://www.zonmw.nl/sites/zonmw/files/2023-05/11.-Mijn-Gender-Wiens-Zorg-rapport-definitief---mei-2023.pdf)

[3] Aitken M, Steensma TD, Blanchard R, et al. Evidence for an altered sex ratio in clinic-referred adolescents with gender dysphoria. J Sex Med 2015 12 756–763. (10.1111/jsm.12817) 25612159

[4] Wood H, Sasaki S, Bradley SJ, et al. Patterns of referral to a gender identity service for children and adolescents (1976–2011): age, sex ratio, and sexual orientation. J Sex Marital Ther 2013 39 1–6. (10.1080/0092623x.2012.675022) 23152965

[5] Nolan IT, Kuhner CJ & Dy GW. Demographic and temporal trends in transgender identities and gender confirming surgery. Transl Androl Urol 2019 8 184–190. (10.21037/tau.2019.04.09) 31380225 PMC6626314

[6] Coleman E, Radix AE, Bouman WP, et al. Standards of care for the health of transgender and gender diverse people, version 8. Int J Transgend Health 2022 23 (Supplement 1) S1–S259. (10.1080/26895269.2022.2100644) 36238954 PMC9553112

[7] Hembree WC. Guidelines for pubertal suspension and gender reassignment for transgender adolescents. Child Adolesc Psychiatr Clin N Am 2011 20 725–732. (10.1016/j.chc.2011.08.004) 22051008

[8] Schagen SE, Cohen-Kettenis PT, Delemarre-van de Waal HA, et al. Efficacy and safety of gonadotropin-releasing hormone agonist treatment to suppress puberty in gender dysphoric adolescents. J Sex Med 2016 13 1125–1132. (10.1016/j.jsxm.2016.05.004) 27318023

[9] Cohen-Kettenis PT, Schagen SE, Steensma TD, et al. Puberty suppression in a gender-dysphoric adolescent: a 22-year follow-up. Arch Sex Behav 2011 40 843–847. (10.1007/s10508-011-9758-9) 21503817 PMC3114100

[10] Rew L, Young CC, Monge M, et al. Review: puberty blockers for transgender and gender diverse youth-a critical review of the literature. Child Adolesc Ment Health 2021 26 3–14. (10.1111/camh.12437) 33320999

[11] de Vries AL, Steensma TD, Doreleijers TA, et al. Puberty suppression in adolescents with gender identity disorder: a prospective follow-up study. J Sex Med 2011 8 2276–2283. (10.1111/j.1743-6109.2010.01943.x) 20646177

[12] Costa R, Dunsford M, Skagerberg E, et al. Psychological support, puberty suppression, and psychosocial functioning in adolescents with gender dysphoria. J Sex Med 2015 12 2206–2214. (10.1111/jsm.13034) 26556015

[13] van der Loos M, Hannema SE, Klink DT, et al. Continuation of gender-affirming hormones in transgender people starting puberty suppression in adolescence: a cohort study in The Netherlands. Lancet Child Adolesc Health 2022 6 869–875. (10.1016/s2352-4642(22)00254-1) 36273487

[14] Brik T, Vrouenraets L, de Vries MC, et al. Trajectories of adolescents treated with gonadotropin-releasing hormone analogues for gender dysphoria. Arch Sex Behav 2020 49 2611–2618. (10.1007/s10508-020-01660-8) 32152785 PMC7497424

[15] Abbruzzese E, Levine SB & Mason JW. The myth of “reliable research” in pediatric gender medicine: a critical evaluation of the Dutch studies-and research that has followed. J Sex Marital Ther 2023 49 673–699. (10.1080/0092623x.2022.2150346) 36593754

[16] Biggs M. The Dutch protocol for juvenile transsexuals: origins and evidence. J Sex Marital Ther 2023 49 348–368. (10.1080/0092623x.2022.2121238) 36120756

[17] Taylor J, Mitchell A, Hall R, et al. Interventions to suppress puberty in adolescents experiencing gender dysphoria or incongruence: a systematic review. Arch Dis Child 2024 109 (Supplement 2) s33–s47. (10.1136/archdischild-2023-326669) 38594047

[18] Cass H. 2024 The Cass Review. Independent review of gender identity services for children and young people: final report. (https://cass.independent-review.uk/home/publications/final-report/)

[19] McNamara M, Lepore C & Alstott A. Protecting transgender health and challenging science denialism in policy. N Engl J Med 2022 387 1919–1921. (10.1056/NEJMp2213085) 36409481

[20] Cheung CR, Abbruzzese E, Lockhart E, et al. Gender medicine and the cass review: why medicine and the law make poor bedfellows. Arch Dis Child 2025 110 251–255. (10.1136/archdischild-2024-327994) 39401844 PMC12013558

[21] Ministry of Health, Welfare and Sport. Kwaliteitsstandaard Transgenderzorg – Somatisch [Quality Standard Transgender Care – Somatic]. The Hague, Netherlands: Ministry of Health, Welfare and Sport, 2018. (https://richtlijnendatabase.nl/uploaded/docs/Kwaliteitsstandaard_Transgenderzorg_Somatisch.pdf)

[22] Federation of Medical Specialists. Overgewicht en obesitas bij volwassenen en kinderen [Diagnostics, support, and care for children with obesity]. Utrecht, Netherlands: Federation of Medical Specialists, 2022. (https://richtlijnendatabase.nl/richtlijn/overgewicht_en_obesitas_bij_volwassenen_en_kinderen/diagnostiek_ondersteuning_en_zorg_voor_kinderen_met_obesitas.html)

[23] Ciancia S, Dubois V & Cools M. Impact of gender-affirming treatment on bone health in transgender and gender diverse youth. Endocr Connect 2022 11 e220280. (10.1530/ec-22-0280) 36048500 PMC9578106

[24] Schagen SEE, Wouters FM, Cohen-Kettenis PT, et al. Bone development in transgender adolescents treated with GnRH analogues and subsequent gender-affirming hormones. J Clin Endocrinol Metab 2020 105 e4252–e4263. (10.1210/clinem/dgaa604) 32909025 PMC7524308

[25] van de Grift TC, van Gelder ZJ, Mullender MG, et al. Timing of puberty suppression and surgical options for transgender youth. Pediatrics 2020 146 e20193653. (10.1542/peds.2019-3653) 33106340

[26] Bessone F, Lucena MI, Roma MG, et al. Cyproterone acetate induces a wide spectrum of acute liver damage including corticosteroid-responsive hepatitis: report of 22 cases. Liver Int 2016 36 302–310. (10.1111/liv.12899) 26104271

[27] Wilson LM, Baker KE, Sharma R, et al. Effects of antiandrogens on prolactin levels among transgender women on estrogen therapy: a systematic review. Int J Transgend Health 2020 21 391–402. (10.1080/15532739.2020.1819505) 34993517 PMC8726721

[28] Angus LM, Nolan BJ, Zajac JD, et al. A systematic review of antiandrogens and feminization in transgender women. Clin Endocrinol 2021 94 743–752. (10.1111/cen.14329) 32926454

[29] McGregor K, McKenna JL, Williams CR, et al. Association of pubertal blockade at Tanner 2/3 with psychosocial benefits in transgender and gender diverse youth at hormone readiness assessment. J Adolesc Health 2023 74 801–807. (10.1016/j.jadohealth.2023.10.028) 38099903

[30] van der Loos M, Vlot MC, Klink DT, et al. Bone mineral density in transgender adolescents treated with puberty suppression and subsequent gender-affirming hormones. JAMA Pediatr 2023 177 1332–1341. (10.1001/jamapediatrics.2023.4588) 37902760 PMC10616766

[31] Lidegaard O, Nielsen LH, Skovlund CW, et al. Venous thrombosis in users of non-oral hormonal contraception: follow-up study, Denmark 2001–2010. BMJ 2012 344 e2990. (10.1136/bmj.e2990) 22577198 PMC3349780

[32] Parkin L, Sharples K, Hernandez RK, et al. Risk of venous thromboembolism in users of oral contraceptives containing drospirenone or levonorgestrel: nested case-control study based on UK general practice research database. BMJ 2011 342 d2139. (10.1136/bmj.d2139) 21511804 PMC3081041

[33] Ciarcia J & Huckins LM. Oral contraceptives and the risk of psychiatric side effects: a review. Complex Psychiatry 2024 10 36–44. (10.1159/000539515) 39148498 PMC11324216

[34] Carmichael P, Butler G, Masic U, et al. Short-term outcomes of pubertal suppression in a selected cohort of 12 to 15 year old young people with persistent gender dysphoria in the UK. PLoS One 2021 16 e0243894. (10.1371/journal.pone.0243894) 33529227 PMC7853497

[35] Khatchadourian K, Amed S & Metzger DL. Clinical management of youth with gender dysphoria in Vancouver. J Pediatr 2014 164 906–911. (10.1016/j.jpeds.2013.10.068) 24315505

[36] Cohen-Kettenis PT & Klink D. Adolescents with gender dysphoria. Best Pract Res Clin Endocrinol Metab 2015 29 485–495. (10.1016/j.beem.2015.01.004) 26051304

[37] Ciancia S, Dubois V, Craen M, et al. Effects of puberty suppression on bone, body composition, handgrip strength and glucolipid profile in early-pubertal transgender adolescents. Int J Transgender Health 2024 26 861–873. (10.1080/26895269.2024.2353224) PMC12312191 40756711

[38] Nielsen CT, Skakkebaek NE, Richardson DW, et al. Onset of the release of spermatozoa (spermarche) in boys in relation to age, testicular growth, pubic hair, and height. J Clin Endocrinol Metab 1986 62 532–535. (10.1210/jcem-62-3-532) 3944237

[39] Hagenäs I, Jørgensen N, Rechnitzer C, et al. Clinical and biochemical correlates of successful semen collection for cryopreservation from 12 to 18-year-old patients: a single-center study of 86 adolescents. Hum Reprod 2010 25 2031–2038. (10.1093/humrep/deq147) 20570975

[40] Fornander MJ, Roberts T, Egan AM, et al. Weight status, medication use, and recreational activities of treatment-naïve transgender youth. Child Obes 2022 18 228–236. (10.1089/chi.2021.0155) 34762510

[41] Keski-Rahkonen A. Eating disorders in transgender and gender diverse people: characteristics, assessment, and management. Curr Opin Psychiatry 2023 36 412–418. (10.1097/yco.0000000000000902) 37781981

[42] Kaltiala-Heino R, Bergman H, Työläjärvi M, et al. Gender dysphoria in adolescence: current perspectives. Adolesc Health Med Ther 2018 9 31–41. (10.2147/ahmt.S135432) 29535563 PMC5841333

[43] Thrower E, Bretherton I, Pang KC, et al. Prevalence of autism spectrum disorder and attention-deficit hyperactivity disorder amongst individuals with gender dysphoria: a systematic review. J Autism Dev Disord 2020 50 695–706. (10.1007/s10803-019-04298-1) 31732891

[44] Connolly MD, Zervos MJ, Barone CJ 2nd, et al. The mental health of transgender youth: advances in understanding. J Adolesc Health 2016 59 489–495. (10.1016/j.jadohealth.2016.06.012) 27544457

[45] Dutch Healthcare Institute/GIPdatabank. Aantal gebruikers naar leeftijd en geslacht per ATC-hoofdgroep N: Zenuwstelsel in 2024 [Total number of prescriptions by age and gender]. Amsterdam, Netherlands: GIPdatabank, 2025. (https://www.gipdatabank.nl/databank?infotype=g&label=00-totaal&tabel_g_00-totaal=B_03-lftgesl&geg=gebr&spec=&item=N)

[46] Central Bureau of Statistics. Bevolking op 1 januari en gemiddeld; geslacht, leeftijd en regio [Population on January 1st and average; gender, age, and region]. The Hague, Netherlands: Central Bureau of Statistics, 2025. (https://opendata.cbs.nl/#/CBS/nl/dataset/03759ned/table?dl=39E0B)

[47] Fahey KML, Kovacek K, Abramovich A, et al. Substance use prevalence, patterns, and correlates in transgender and gender diverse youth: a scoping review. Drug Alcohol Depend 2023 250 110880. (10.1016/j.drugalcdep.2023.110880) 37480799

[48] van der Loos M, Klink DT, Hannema SE, et al. Children and adolescents in the Amsterdam cohort of gender dysphoria: trends in diagnostic- and treatment trajectories during the first 20 years of the Dutch protocol. J Sex Med 2023 20 398–409. (10.1093/jsxmed/qdac029) 36763938

[49] Kaltiala-Heino R, Sumia M, Työläjärvi M, et al. Two years of gender identity service for minors: overrepresentation of natal girls with severe problems in adolescent development. Child Adolesc Psychiatry Ment Health 2015 9 9. (10.1186/s13034-015-0042-y) 25873995 PMC4396787

[50] Arnoldussen M, de Rooy FBB, de Vries ALC, et al. Demographics and gender-related measures in younger and older adolescents presenting to a gender service. Eur Child Adolesc Psychiatry 2023 32 2537–2546. (10.1007/s00787-022-02082-8) 36370316 PMC10682114

[51] Ashley F. Shifts in assigned sex ratios at gender identity clinics likely reflect changes in referral patterns. J Sex Med 2019 16 948–949. (10.1016/j.jsxm.2019.03.407) 31053562

[52] de Vries AL, Steensma TD, Cohen-Kettenis PT, et al. Poor peer relations predict parent- and self-reported behavioral and emotional problems of adolescents with gender dysphoria: a cross-national, cross-clinic comparative analysis. Eur Child Adolesc Psychiatry 2016 25 579–588. (10.1007/s00787-015-0764-7) 26373289 PMC4889630

