# Supplementary material for: GnRH analogs as a monotherapy in transgender and gender-diverse adolescents: clinical insights from a single-center study
Source: Endocr Connect. 2025 Aug 14;14(8):e250292. doi: 10.1530/EC-25-0292 (PMC12358825; doi:10.1530/EC-25-0292)
Supplement: Supplementary file 1 [file supplementary_materials.pdf]

Supplemental Tables

Other laboratory tests comparing Tanner 2/3 with Tanner 4/5 in AFAB at T0

		N	Mean	SD	p
Hemoglobin (mmol/L)	T 2/3	29	8,10	,46	**.04**
	T 4/5	36	8,40	,67	
Hematocrite (L/L)	T 2/3	29	,39	,025	**.03**
	T 4/5	36	,41	,027	
Leukoctyes (10**9/L)	T 2/3	29	6,48	1,31	.40
	T 4/5	36	6,81	1,77	
Thrombocytes (10**9/L)	T 2/3	29	294,31	50,95	.67
	T 4/5	36	287,42	74,33	
Urea (mmol/L)	T 2/3	27	4,06	,85	**.04**
	T 4/5	34	3,57	,93	
Creatine (µmol/L)	T 2/3	27	46,89	7,33	**<.001**
	T 4/5	34	56,59	8,39	
ALAT (U/L)	T 2/3	28	19,77	6,27	.14
	T 4/5	33	17,18	7,23	
ASAT (U/L)	T 2/3	28	27,46	6,0948	**.001**
	T 4/5	34	21,62	7,24	
Lactate Dehydrogenase (U/L)	T 2/3	28	226,18	36,89	**<.001**
	T 4/5	34	181,91	39,38	
GammaGT (U/L)	T 2/3	28	10,86	1,90	**.04**

	T 4/5	34	13,76	7,08	
Vitamin D (nmol/L)	T 2/3	28	63,64	17,18	**.002**
	T 4/5	35	48,26	19,61	

Other laboratory tests comparing Tanner 2/3 with Tanner 4/5 in AMAB at T0

		N	Mean	SD	p
Hemoglobin (mmol/L)	T 2/3	18	8,38	,42	**.009**
	T 4/5	12	8,97	,73	
Hematocrite (L/L)	T 2/3	18	,40	,019	**.003**
	T 4/5	12	,43	,026	
Leukoctyes (10**9/L)	T 2/3	18	6,19	1,48	.76
	T 4/5	12	6,36	1,54	
Thrombocytes (10**9/L)	T 2/3	18	288,33	65,04	.12
	T 4/5	12	254,42	42,06	
Urea (mmol/L)	T 2/3	18	3,74	,99	.65
	T 4/5	12	3,93	1,14	
Creatine (µmol/L)	T 2/3	18	46,83	6,87	**<.001**
	T 4/5	12	62,33	13,11	
ALAT (U/L)	T 2/3	18	16,22	4,49	.14
	T 4/5	12	19,25	6,40	
ASAT (U/L)	T 2/3	18	27,11	11,0288	.24
	T 4/5	12	22,912	5,55	

Lactate Dehydrogenase (U/L)	T 2/3	18	225,00	24,32	**.04**
	T 4/5	12	200,92	38,19	
GammaGT (U/L)	T 2/3	18	12,83	3,13	**.04**
	T 4/5	12	15,67	3,94	
Vitamin D (nmol/L)	T 2/3	18	65,61	21,28	.11
	T 4/5	11	52,82	18,78	
